# Fencing injuries in French elite fencers: a retrospective analysis from 2016 to 2023

**DOI:** 10.3389/fspor.2025.1535818

**Published:** 2025-05-30

**Authors:** Hugo Gondouin, Nicolas Zabotto, Edem Allado, Oriane Hily, Margaux Temperelli, Sébastien Le Garrec, Bruno Chenuel, Mathias Poussel

**Affiliations:** ^1^ CHRU-Nancy, University Centre of Sports Medicine and Adapted Physical Activity, Université de Lorraine, Nancy, France; ^2^ DevAH, Department of Physiology, Université de Lorraine, Nancy, France; ^3^ Medical Commission, French Fencing Federation, Noisy le Grand, France; ^4^ Institut National du Sport, De l’Expertise et de la Performance (INSEP), Paris, France

**Keywords:** athletic injury, sport medicine, fencing, elite athlete, Olympic

## Abstract

**Background:**

Fencing is one of the five sports that have been permanent fixtures at the Olympic Games since the first modern Games in 1896. However, the available literature on fencing-related injuries is very scarce, even more so for elite fencers. This study aimed to assess injuries in elite French fencers to more precisely characterize the injury patterns in this Olympic sport.

**Design:**

The study included all elite fencers from the French National Institute for Sport, Expertise and Performance from June 2016 to May 2023. Injuries were recorded using the medical information system, which documents any newly incurred injury. Each injury was specified and then stratified according to weapon category (epee, foil, and saber), sex, injury location, and types of anatomical structures involved.

**Results:**

A total of 117 different fencers (female = 56) were included, and 1,470 injuries were recorded for an overall injury rate of 2.55 injuries/year/fencer (female = 2.61; male = 2.50; NS, non-significant), mostly occurring in the lower limbs (71%). Epee fencers show more knee (*p* = 0.046) and forearm (*p* = 0.020) injuries and less thigh injuries (*p* < 0.005), as well as more injuries involving joints (*p* = 0.026) but less muscle injuries (*p* = 0.005). Females showed more injuries located in the pelvis and hip (10% vs. 5.3%; *p* = 0.001).

**Conclusions:**

Our study confirms a low overall injury rate in elite fencers and highlights some particularities for epee compared with the other two conventional weapons, in addition to sex-specific differences concerning injury location.

## 1 Introduction

Since the very beginning of the modern era of the Olympic Games (i.e., 1896), fencing has always been represented. Initially, only included for men (foil and saber from 1896 and epee from 1900), it was not until 1924 that women's Olympic competitions were introduced (foil from 1924, epee from 1996, and saber from 2004). The very first fencing Olympic gold medal was won by the French fencer Eugène-Henri Gravelotte (1876–1939), thus inaugurating a long French tradition of high-level fencing. Since then, fencing has become France's leading source of Olympic medals with a total of 130 awards, and even today, France remains one of the major nations in this sport, with a significant number of elite athletes.

Fencing is an open-skill opposition sport usually classified with a low static component and a moderate dynamic component or more recently as a mixed (i.e., combination of skill and power components) and high-intensity sport (1, 2). Overall characteristics of fencing have been previously reviewed, showing a high physical demand but also specific skills regarding technique, tactics, and perceptual and psychomotor abilities. Although fencing remains an amateur sport (i.e., non-professional), it has undergone major changes during the last decades with a continuous increase in physical, technical, and psychological demands for athletes, in a context of a constant quest for performance (3).

Compared to other sports (4, 5), available literature dealing with reports of fencing injuries is very scarce, and it is quite difficult to outline a specific epidemiology (3, 6, 7). Although fencing did not appear as particularly dangerous (keeping in mind that protective equipment standards and blade quality continuously increased), there is potentially a fine line between a fatal puncture wound (8) and a minor injury from a broken blade (9). The design also varies considerably from one study to another (with variable levels of practice, duration, or definitions of injuries) making comparisons difficult (10). However, considering the specificity of each weapon category (i.e., epee, foil, and saber) and their completely different styles of play, strategy, and tactics, it is entirely conceivable that injury patterns differ. Only a few studies involved elite fencers, but Park and Brian Bung (11) showed some interesting findings in their 8-year follow-up study from a sample of elite Korean fencers. Indeed, they found an injury rate per athlete of 3.3 injuries/year, mostly concerning the lower extremity (47.2%). They also highlighted, for all three weapons, significant differences between female and male fencers in terms of location and severity of injuries.

Overall, considering the lack of available data on fencing injuries, especially among elite fencers, our study aimed to describe and analyze the fencing injuries of top French elite fencers during a period of almost three Olympiads. We hypothesize that weapon and sex-specific differences potentially exist regarding fencing injuries.

## 2 Materials and methods

### 2.1 Participants

The study included all elite fencers who trained at the French National Institute for Sport, Expertise and Performance [Institut National du Sport, de l'Expertise et de la Performance (INSEP), France]. This national training center is dedicated to the very top French athletes and to prepare them for international competitions. Fencers were retrospectively included during a period of almost the last three Olympic Games, from June 2016 to May 2023. An average of 24 elite fencers were annually accepted to train in the center for each weapon category, with a minimum and maximum group size of 69 and 78 fencers/year, respectively. French elite fencers trained an average of 24 h/week for 11 months a year for an overall of 1,056 h/year. In addition, participation in competitions throughout the fencing season could be estimated to 32 days (12 days of national contests and 20 days of international competitions).

### 2.2 Data collection

Data collection was performed using the MAIDIS medical information system (MAIDIS SAS, France) used at the French training center. This medical information system logs all data regarding the characteristics of each fencer (sex, weapon), and a specific injury report form is created and filled out at any newly incurred fencing injury. For each injury form, characteristics of the injury are specified (body part, type of anatomical structures involved) (12). The MAIDIS system has been exclusively filled by a member of the French medical team (sports medicine physician). Sports injury was defined as any musculoskeletal sign (acute or overuse) and concussion occurring during fencing practice (training and competition), regardless of the consequences of a possible interruption of sports activities (13, 14). When a single injury incident affects several different anatomical structures, each anatomical structure is recorded separately. Data were then stratified according to the weapon category (epee, foil, and saber), sex, injury location, and types of anatomical structures involved.

### 2.3 Statistical analysis

Both descriptive and comparative analyses were made by accounting for the nature and distribution of the variables. Qualitative variables were described as frequencies and percentages, whereas quantitative variables with normal distribution were evaluated using the mean ± standard deviation (SD) and quantitative variables with non-parametric distribution with the median and interquartile range (IQR). The chi-square test or Fisher's exact test, with, if necessary, the exact calculation of Fisher, was used for the ordinal or nominal data analysis. The significance level was set at 0.05 for the entire study. IBM SPSS Statistics 23.0 was used for the data analysis.

## 3 Results

During the study period (7-year follow-up), a total of 117 elite fencers (19 left-handed, 16.3%; 98 right-handed, 83.7%) were included (with an average attendance to the INSEP of 5.31 years), and a total of 1,470 fencing injuries were recorded (Table 1). Among them, 767 injuries occurred in females (*n* = 56) and 703 in males (*n* = 61; NS). Overall, 556 fencing injuries (37.8%) occurred in epee, 464 (31.6%) in foil, and 450 (30.6%) in saber.

**Table 1:** Number of fencers included during the 7-year follow-up, according to gender and weapon categories. Data are presented as *n* (%) for dichotomous variables.

Weapon Gender	Epee (*n* = 34)	Foil (*n* = 41)	Saber (*n* = 42)	Total
Male	19 (55.9)	20 (48.8)	22 (52.4)	61
Female	15 (44.1)	21 (51.2)	20 (47.6)	56

### 3.1 Fencing injury rates (IR)

Overall fencing injury rate was 2.55 injuries/year/fencer (female = 2.61; male = 2.50; NS). Among the three weapons, IR = 2.84 injuries/year/fencer for epee (female = 2.55; male = 3.08), IR = 2.53 injuries/year/fencer for foil (female = 2.59; male = 2.48), and IR = 2.34 injuries/year/fencer for saber (female = 2.67; male = 2.03). Considering an average fencing practice of 1,056 h/year/fencer, overall IR was 2.41 injuries/1,000 h of training (female = 2.47; male = 2.37; NS). Among the three weapons, IR = 2.69 injuries/1,000 h of training for epee (female = 2.41; male = 2.92), IR = 2.40 injuries/1,000 h of training for foil (female = 2.45; male = 2.35), and IR = 2.22 injuries/1,000 h of training for saber (female = 2.53; male = 1.92). We did not find any difference in IR between weapons or between sex within each weapon.

### 3.2 Fencing injury location and anatomical structures involved

The lower extremity was the predominant location affected, with 71% of the injuries, followed by the upper extremity (18.4%) and trunk (10.4%). Among the 1,044 lower extremity injuries, 60.6% concerned the front limb and 39.4% the rear limb. Among the 269 upper extremity injuries, 96.3% concerned the dominant arm. Fencing injuries locations, according to weapon categories, are presented in Table 2. Compared to foil and saber, epee showed more knee (*p* = 0.046) and forearm (*p* = 0.020) injuries and less thigh injuries (*p* < 0.005). Compared to epee and saber, foil showed less foot injuries (*p* = 0.050).

**Table 2:** Fencing injury location (body region and site) in elite French fencers according to the three weapons (epee, foil, and saber) (12).

Location	Epee	Foil	Saber	Total	*p*-value*
Head	0 (0)	2 (0.4)	1 (0,2)	3 (0.2)	0.276
Trunk	71 (12.8)	42 (9.0)	41 (9.2)	154 (10.4)	
• Thoraco-lombar spine	59 (10.6)	34 (7,3)	34 (7,6)	127 (8,6)	0.177
• Chest	12 (2.2)	8 (1.7)	7 (1.6)	27 (1.8)	0.645
Upper limb	123 (22.2)	77 (16.6)	69 (15.3)	269 (18.4)	
• Shoulder	31 (5.6)	17 (3.7)	15 (3.3)	63 (4.3)	0.233
• Upper arm	7 (1.3)	8 (1.7)	8 (1.8)	23 (1.6)	0.644
• Forearm	18 (3.2)	3 (0.6)	8 (1.8)	29 (2.0)	0.020
• Hand/wrist	67 (12.1)	49 (10.6)	38 (8.4)	154 (10.5)	0.240
Lower limb	361 (65)	344 (74.1)	241 (75.1)	1,044 (71)	
• Hip/groin	37 (6.7)	45 (9.7)	32 (7.1)	114 (7.8)	0.244
• Thigh	72 (12.9)	125 (26.9)	101 (22.4)	298 (20.3)	<0.005
• Knee	125 (22.5)	78 (16.8)	74 (16.4)	277 (18.8)	0.046
• Lower leg	21 (3.8)	20 (4.3)	24 (5.3)	65 (4.4)	0.501
• Achilles	18 (3.2)	4 (0.9)	10 (2.2)	32 (2.2)	0.052
• Ankle	52 (9.4)	53 (11.4)	60 (13.3)	165 (11.2)	0.208
• Foot	36 (6.5)	19 (4.1)	38 (8.4)	93 (6.3)	0.050

Data are presented as *n* (%) for dichotomous variables. NB, an injury can involve multiple locations.

* We used a chi-square test for variables by weapon category.

Overall, elite female fencers showed more fencing injuries located in the pelvis and hip (10% vs. 5.3%; *p* = 0.001). No other sex difference has been shown regarding injury location. Within each weapon category, our results also found some differences between females and males in the injury location. Females showed more fencing injuries located to the pelvis and hip for epee (10.8% vs. 2.2%; *p* = 0.024) and for foil (12.9% vs. 6.7%; *p* < 0.005). For foil, females showed more injuries located on the foot (6.2% vs. 2.1%; *p* = 0.023).

Table 3 presents the various anatomical structures involved (overall and weapon distribution). Among the 1,470 fencing injuries, we could identify the tissue involved in 1,170 cases. Compared to foil and saber, epee showed more injuries involving joints (*p* = 0.026) but less muscle injuries (*p* = 0.005). Compared to epee and saber, foil showed less ligament injuries (*p* = 0.043). We did not observe any difference between weapons concerning tendon injuries (NS). Overall, elite male fencers showed more tendon injuries (24.2% vs. 18.8%; *p* = 0.019) and elite female fencers more joint injuries (24% vs. 15.9%; *p* < 0.005). No difference between females and males has been shown regarding ligament and muscle injuries (NS). Within each weapon category, our results found some differences between females and males in terms of the anatomical structures involved. For epee, females showed more muscle injuries (15.7% vs. 8.9%, *p* = 0.024). For foil, females showed more joint injuries (26.7% vs. 10.9%; *p* < 0.005), whereas males showed more tendon injuries (28% vs. 16.4%; *p* = 0.004).

**Table 3:** Anatomical structures involved in French elite fencers regarding weapon categories (epee, foil, and saber) (12).

Tissue	Epee	Foil	Sabre	Total	*p*-value*
Muscle	69 (12.4)	91 (19.6)	88 (19.6)	248 (16.9)	0.005
Joint	134 (24.1)	86 (18.5)	76 (16.9)	296 (20.1)	0.026
Ligament	69 (12.4)	43 (9.3)	69 (15.3)	181 (12.3)	0.043
Tendon	132 (23.7)	104 (22.4)	78 (17.3)	314 (21.4)	0.069
Superficial tissues/skin	4 (0.7)	6 (1.3)	1 (0.2)	11 (0.7)	0.215
Others	41 (7.4)	34 (7.3)	45 (10.0)	120 (8.2)	0.314
Missing data	121 (21.8)	119 (25.6)	114 (25.3)	354 (24.1)	0.336

Data are presented as *n* (%) for dichotomous variables. NB, an injury can affect multiple types of tissues.

* We used a chi-square test for variables by weapon category.

## 4 Discussion

Only a few studies are available regarding injuries in fencing, and even fewer deal with top elite fencers or have a weapon-specific concern. Our major result is the overall fencing injury rate of 2.55 injuries/year/fencer or 2.41 injuries/1,000 h of training in French elite fencers, mostly occurring in the lower limbs (71%). Although fencing remains a non-professional sport, it has undergone major developments since its first representation at the Olympic Games in 1896, with significant specialization depending on the weapon practiced. If some fencers were previously able to compete at the highest level in the three weapons (as Lucien Gaudin, 1886–1934; seven Olympic medals in epee, foil, and saber), it is now no longer possible because of the specificities and particularities inherent to each weapon. Indeed, epee fencing is mainly slow-paced, whereas foil and saber are faster-paced and more explosive. Thus, training loads, exercise types, technique, and strategy are very different between the three weapons. Our results may therefore help to better understand the pattern of fencing injuries in elite fencers.

Our injury rate of 2.55 injuries/year/fencer or 2.41 injuries/1,000 h of training in French elite fencers is slightly lower compared with (but still quite comparable) the results of Park and Brian Byung (11) showing an IR of 3.3 injuries/year/fencer or 3.00 injuries/1,000 h of training in Korean elite fencers (also mainly concerning the lower extremity but for only 47.2%). To our knowledge, even if some other studies deal with elite fencers, the study of Park and Brian Byung is the only one that allows a significant comparison (11, 15, 16). Indeed, in both studies, elite fencers trained in a national training center, with a similar average of training time (i.e., 1,073 h/year for Korean elite fencers vs. 1,056 h/year for French elite fencers). Moreover, fencing injuries were rigorously assessed by sports medicine physicians, during a comparable period of almost 7 years. If the fear of a fatal puncture wound from a broken blade is still omnipresent in fencing, it appears that injuries are mostly minor and IR among the lowest compared with other sports, such as soccer or basketball (with an IR of 50 and 31 times higher than fencing, respectively) (6, 15). Our results therefore support a low IR that appears similar between French and Korean elite fencers. In addition, we also show that lower limbs are mostly affected (71%) in fencing supporting previous studies even if some other older studies initially found a higher proportion of injuries affecting upper extremities (3, 6, 11).

Considering overall fencing injuries locations, even if we found a greater proportion of lower limb injuries, we show a similar distribution of injury location for our French elite fencers compared with Korean elite fencers for the three major body regions affected: 71% for lower extremities (vs. 47.2%), 18.4% for upper extremities (vs. 26.4%), and 10.4 for trunk (vs. 21.4%). Beyond these body area categories, we however could also show some slight differences. Indeed, the three most sites damaged were thigh (20.3%), knee (18.8%), and ankle (11.2%) for French elite fencers, whereas the three most sites impaired were ankle (11.4%), knee (10.1%), and lumbar spine/lower back (9.8%) for Korean elite fencers (12). Considering the anatomical structures most frequently involved, we also found comparable results to available literature with tendon, joint, and muscle affected for 21.4%, 20.1%, and 16.9%, respectively (6, 11, 16). Our results are thus in line with scarce data available and allow us to better characterize the epidemiology of fencing injuries in high-level fencers.

The three fencing weapons have different compositions, techniques, and scoring target areas, and foil and saber are considered conventional weapons because of a “right of way” point awarded system. Therefore, a focus on fencing injuries according to weapon categories may be helpful to further characterize the specificities of fencing. Our results showed more knee (*p* = 0.046) and forearm (*p* = 0.020) injuries and less thigh injuries (*p* < 0.005) for epee fencers (Table 2). Considering the anatomical structures involved (Table 3), epee showed more injuries involving joints (*p* = 0.026) but less muscle injuries (*p* = 0.005). Combined with the highest IR of 2.84 injuries/year/fencer, our results support some specificities for epee that is the only weapon with no “right of way” in operation and in which the whole body (including hand and foot) is a scoring area. The physical and technical skills, strategies, and tactics of epee fencing are therefore very specific and quite different from those of the other two conventional weapons. Assault times and the time between touches are by far the longest, and some actions are very specific to epee fencing (e.g., toe touches) (17). All this may explain the different pattern of fencing injuries in epee. Interestingly, Park and Brian Byung (11) did not find any particularities for epee, but showed a higher IR for saber (IR = 3.9 injuries/year/fencer) and also some particularities and differences between females and males in saber. One explanation of this difference could lie in the difference between the tradition of fencing in Europe and the more recent history of fencing in other nations. For instance, the fencing technique of Korean fencers is mainly based on speed with fast footwork and explosive lunge, whereas European and French fencers mostly express a fencing style based on strong hand technique and strength. Another explanation could also be related to the “preferred” weapon of a nation. Indeed, saber appears to be the preferred weapon for Korean fencing. Among the 17 Olympic medals won by Korean fencers, 9 (53%) were obtained in saber. Similarly, epee appears to be the preferred weapon in France, with 42% of Olympic medals won in epee for French fencers (of the 130 Olympic medals won, 55 were in epee). This could support that although the overall IR in elite fencing is low, the higher the level of fencers in a weapon, the higher the incidence of fencing injury.

Even if we did not find any difference in the overall IR between sexes, the comparison between females and males highlights some differences. Indeed, females showed more injuries located in the pelvis and hip (10% vs. 5.3%; *p* = 0.001). Males showed more tendon injuries (24.2% vs. 18.8%; *p* = 0.019) and females more joint injuries (24% vs. 15.9%; *p* < 0.005). Females showed more injuries located in the pelvis and hip for epee (10.8% vs. 2.2%; *p* = 0.024) and for foil (12.9% vs. 6.7%; *p* < 0.005). Our results thus show a discrepancy with other available studies that showed either a higher IR for men (for saber and foil) or for women (11, 18). However, French elite female fencers appear comparable to US national fencing team members and US fencing Olympians also showing more hip injuries (16). No doubt that the differences in physical build and physiological characteristics between females and males play a major role, leading to different training strategies and fencing techniques, but it is not clear how it impacts the IR in both sexes.

Our results also have some limitations that should be highlighted. Since our first goal was to clarify the main characteristics (type, location, frequency) of the most common fencing injuries in elite athletes, we did not precisely study their severity. However, it has been accepted in available general recommendations or other more specific literature regarding fencing that an operational definition for an injury event should include the duration of time loss related to the injury (12). The duration of time loss could then easily reflect the severity of the injury, allowing a stratification following different time bins (time loss of 0 days, 1–7 days, 8–28 days, and >28 days). The definition we used in our study did not include such a time loss criterion because it was not available in the medical information system. We therefore could not clearly distinguish injuries that impact sports participation (i.e., with time loss) from those that did not impact participation (i.e., without time loss). On the other hand, it has also been accepted that using the time loss is imperfect to measure the severity of injuries (International Olympic Committee consensus). Overall, the lack of this criterion in our definition is undoubtedly a limitation insofar as it allows a lesser degree of comparison with other sports epidemiology studies.

All the injuries reported in the study occurred during fencing, therefore allowing us to have a specific focus on fencing injuries; however, we did not have further precision on whether these injuries occurred during on-piste training or competition. We thus could not be able to highlight any difference in IR between training and competition. Moreover, we were unable to completely follow available guidelines (12) for recording and reporting of epidemiological data on injury in sport because some information of interest was not available. For instance, if we could present the location and anatomical structure involved, we could not precisely determine the pathology type. These are also limitations of our study because it should have been informative for fencers, coaches, and medical staff.

Finally, as fencing is a one-sided sport, it results in asymmetrical movements, the repetitions of which (training and competition) may lead to asymmetric anthropometrical characteristics. One of the key actions in fencing is the one-sided lunge, where the upper limb movement is immediately followed by the forward knee extensor muscles contraction (dominant leg) and the lengthening of the back leg (non-dominant leg). Fencers have been shown to have a greater cross-sectional area of the dominant forearm and arm (19) but also asymmetric characteristics for the lower limbs (3). Therefore, it is frequently discussed how this asymmetry of the limb could also impact the injuries suffered by fencers (20). Unfortunately, our study was not designed to highlight such potential correlation, thus constituting a further limit that should be addressed in future studies.

## 5 Conclusion

Fencing belongs to the five sports that have been permanent fixtures at the Olympic Games since the first modern Games in 1896. Scarce literature is available regarding injuries in fencing, even fewer dealing with elite fencers, resulting in a lack of precise epidemiology of fencing injuries and making it difficult to develop any strategy of prevention. Our study confirms a low overall injury rate of 2.55 injuries/year/fencer in French elite fencers, mostly occurring in the lower limbs (71%), coupled with some particularities for epee compared with the two other conventional weapons. Our results also suggest sex-specific differences concerning injury location. Considering the paucity and the various methodological designs of available studies, our results allow us to more precisely characterize fencing injuries in elite fencing. Further studies should be helpful to better highlight the specificities of each weapon, between females and males, and also maybe between different styles of fencing to implement specific prevention programs, keeping in mind the asymmetrical nature of fencing, which necessarily requires special attention.

## Data Availability

The raw data supporting the conclusions of this article will be made available by the authors, without undue reservation.

## Practical Implications

• French elite fencers show a low overall injury rate
• Female French elite fencers show more pelvis and hip injuries than male
• Some particularities have been found for epee compared to sabre and foil (conventional weapons)
